# Androgen receptor inhibition sensitizes glioblastoma stem cells to temozolomide by the miR-1/miR-26a-1/miR-487b signature mediated WT1 and FOXA1 silencing

**DOI:** 10.1038/s41420-025-02517-6

**Published:** 2025-05-21

**Authors:** Ana Belén Díaz Méndez, Marta Di Giuliani, Andrea Sacconi, Elisa Tremante, Valentina Lulli, Marta Di Martile, Giulia Vari, Francesca De Bacco, Carla Boccaccio, Giulia Regazzo, Maria Giulia Rizzo

**Affiliations:** 1 Department of Research, Advanced Diagnostics and Technological Innovation, Translational Oncology Research Unit, IRCCS Regina Elena National Cancer Institute, Rome, Italy; 2 Clinical Trial Center, Biostatistics and Bioinformatics Unit, IRCCS Regina Elena National Cancer Institute, Rome, Italy; 3 Department of Oncology and Molecular Medicine, Istituto Superiore di Sanità, Rome, Italy; 4 Preclinical Models and New Therapeutic Agents Unit, IRCCS Regina Elena National Cancer Institute, Rome, Italy; 5 PhD Program in Molecular Medicine, Department of Molecular Medicine, Sapienza University of Rome, Rome, Italy; 6 Laboratory of Cancer Stem Cell Research, Candiolo Cancer Institute, FPO-IRCCS, Candiolo, Italy; 7 Department of Oncology, University of Torino, Turin, Italy

**Keywords:** Cancer stem cells, Cancer epigenetics

## Abstract

Glioblastomas (GBMs) are aggressive brain tumors and challenging cancers for diagnosis and treatment. Therapeutic options include surgery followed by chemotherapy with the DNA alkylator temozolomide (TMZ) and radiotherapy. However, the patient's prognosis remains poor due to tumor heterogeneity, cell infiltration and intrinsic or acquired resistance to therapy. Understanding the resistance mechanisms together with identifying new biomarkers are crucial for developing novel therapeutic strategies. MiRNAs play an important role in the biology of gliomas, they modulate tumorigenesis and therapy response. We recently identified the diagnostic/prognostic miR-1-3p, miR-26a-1-3p and miR-487b-3p signature that displays an oncosuppressive role on several glioma biological functions. In this study, we investigated the effects of the therapeutic potential of this three-miRNA signature as a regulator of response to TMZ. We found that ectopic expression of the miRNA signature in patient-derived GBM neurospheres treated with TMZ impaired cell proliferation and viability by necroptosis induction. Moreover, we identified WT1 and FOXA1, two transcription factors specifically involved in TMZ resistance, as novel direct targets of the miRNA signature. Of note, the repression of WT1 and FOXA1, elicited by the signature, caused a downregulation of the Androgen Receptor (AR) expression, an impairment of tumor-spheroid formation and reversed cancer cell stemness. These results were recapitulated using the AR inhibitor enzalutamide, confirming the involvement of the AR pathway. Our data indicate that the miR-1-3p/miR-26a-1-3p/miR-487b-3p signature, which has an impact on treatment response and cell stemness, may pave the way for miRNA-based complementary therapies in GBM patients.

## Background

Glioblastoma (GBM) is the most prevalent and aggressive type of glioma, accounting for half of all malignant brain tumors. Prognosis in patients with GBM remains extremely poor, with a 5-year survival of 2–10% [1]. Among the main prognostic factors there are mutations of IDH genes that are linked to a better prognosis and are essential from a diagnostic perspective as, according to the World Health Organization (WHO) 2021 classification, all IDH-wild-type gliomas allow to diagnose “glioblastoma IDH-wild-type CNS WHO grade 4” even in the absence of a glioblastoma histopathology [2]. GBMs represent a medical challenge due to their anatomical location, diffuse, infiltrative growth, the resulting impact on brain functioning and their biological complexity [3]. The clinical management of GBM includes surgical resection, followed by a combination of chemotherapy and regional fractionated ionizing radiation [4]. Temozolomide (TMZ), an alkylating agent, is the most preferred and approved drug for either first- or second-line chemotherapy in GBM patients. However, the majority of patients do not respond to therapies due to the intrinsic or acquired ability of GBM cells to develop chemoresistance, and nearly all of them ultimately experience a recurrence or a progression of the disease [4]. An important TMZ-response predictive marker is the O6-methylguanine-DNA methyltransferase (MGMT) methylation status. MGMT activity and the presence of uniquely resistant populations of glioma stem cells are the major contributors to TMZ resistance and the main reasons for treatment failure [5, 6]. Indeed, MGMT-unmethylated (MGMT-unmet) patients are resistant to TMZ and have a much shorter survival compared to those with MGMT methylation (MGMT-met) [7]. Strategies to overcome inherent and acquired resistance to TMZ in GBM have been investigated [6, 7]. However, there have been no successful treatments to render MGMT-unmet GBMs susceptible to TMZ, and therefore, there is an urgent need for novel treatment strategies, especially for this subgroup of patients [7].

MicroRNAs (miRNAs), small non-coding RNAs with epigenetic functions, have been found to play a critical role in the biology of gliomas and in their cancer response to treatment [8–10]. Several miRNAs have been reported as modulators of response to chemotherapy and radiotherapy (RT), either acting as sensitizers or promoters of drug resistance [11, 12]. Recently, we have shown that a diagnostic and prognostic three-miRNA signature (miR-1-3p, miR-26a-1-3p, and miR-487b-3p, from here miR-1/-26a-1/-487b) is differentially expressed in the serum of glioma patients according to IDH genes mutation status, correlating with both patient Overall and Progression Free Survival. We also found that all the miR-1/-26a-1/-487b signature members, and notably their combination, display oncosuppressive functions in GBM cells, impacting cell proliferation, migration and invasion [13]. While miR-1 has been described as a tumor suppressor in many cancers, including gliomas [14–17], as well as a sensitizing agent in chemotherapy and RT [18], scarcer information is available for miR-487b [19] and especially for miR-26a-1-3p. In fact, for this last miRNA, the 5p strand is the mature form mainly investigated [20–22]. In this study, we investigated the therapeutic potential of miR-1/-26a-1/-487b signature as a regulator of response to TMZ treatment in GBM. We found that the miRNA signature inhibits proliferation with a concomitant increase in cell mortality triggered by necroptosis upon TMZ treatment. Furthermore, we have identified WT1 and FOXA1 genes, two transcription factors specifically involved in TMZ response, as novel and direct targets of miR-1/miR-26a-1 (WT1) and miR-487b (FOXA1). Notably, overexpression of the miRNA signature downregulated the Androgen Receptor (AR) expression via WT1 and FOXA1 repression, impaired the tumor-sphere forming ability of cells and was able to reverse cancer stemness. Thus, these data support a potential application of the miR-1/-26a-1/-487b signature and its targets for novel combined therapeutic interventions in GBM.

## Results

### miR-1/-26a-1/-487b signature sensitizes neurospheres to TMZ

**Fig. 1:**
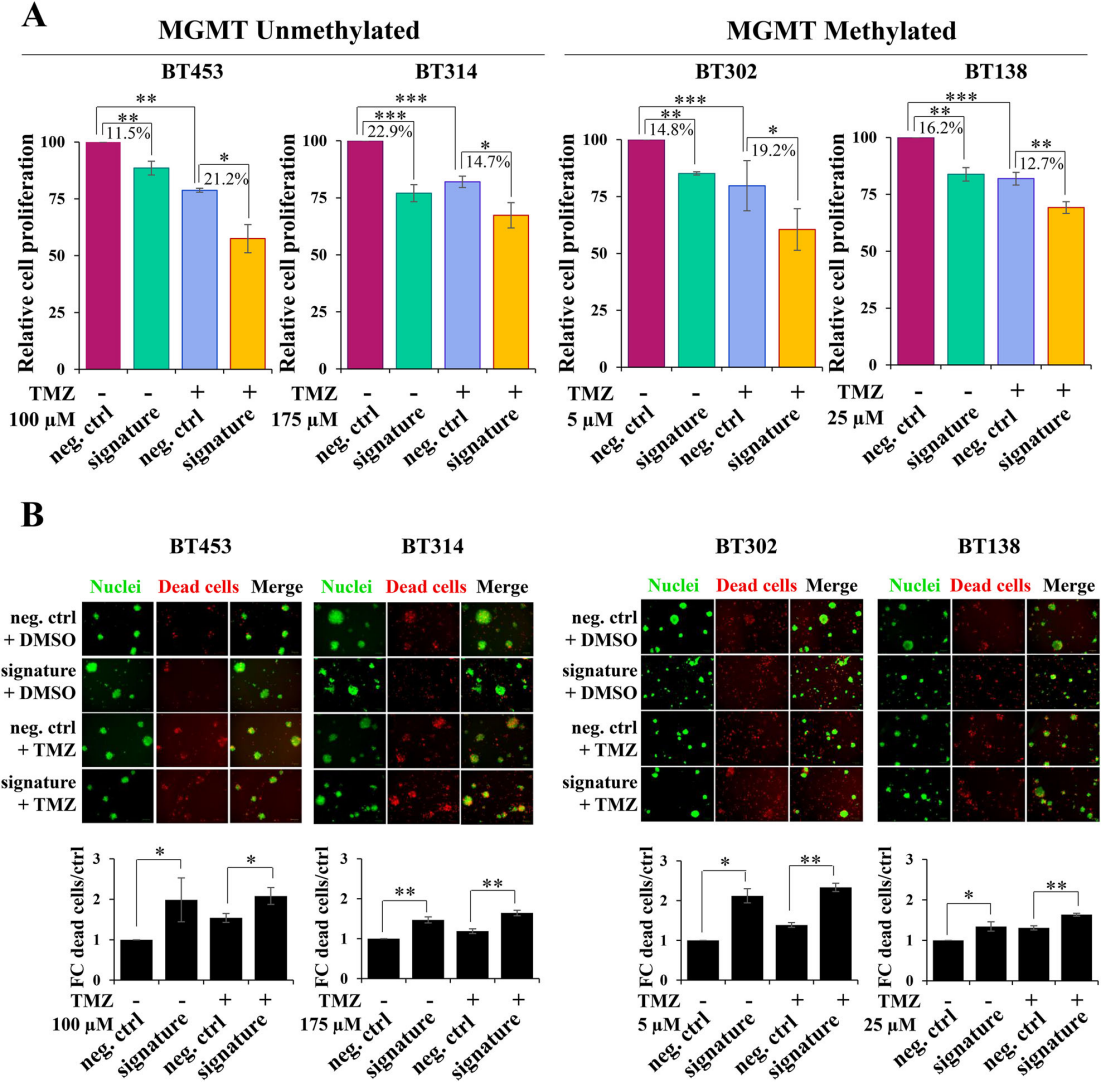
MiRNA signature affects cell proliferation and viability after TMZ treatment. Analysis of **A** cell proliferation by MTT and **B** viability by Cyto3D™Live-Dead assay in the indicated GBM neurospheres overexpressing the miRNA signature mimics or negative control mimic (neg. ctrl) after 6 days of TMZ treatment with the indicated doses. Cell proliferation values are expressed as optical density at 750 nm wavelength, proportional to the quantity of metabolically active cells and viability as fold change of dead cells respect to control. All the values are reported as the mean of at least three experiments. Error bars indicate the standard deviation. *p ≤ 0.05, **p ≤ 0.01, ***p ≤ 0.001.

To study the involvement of the three-miRNA signature in TMZ treatment response in GBM, we evaluated the effects of its overexpression during treatment in MGMT-unmet and MGMT-met GBM neurospheres. To identify the optimal TMZ dose for the analyses, we took into account that the signature alone produced around 20–30% reduction of cell proliferation (Fig. 1A), therefore, we performed dose-response curves to select an appropriate IC20 TMZ dose for each GBM neurosphere model (BT453: 100 µM; BT314: 175 µM; BT302: 5 µM; BT138: 25 µM; Supplementary Fig. S1).

As shown in Fig. 1A, B, we found that, after TMZ treatment, the miRNA signature ectopic expression decreased proliferation and increased cell mortality compared to negative control mimics, although not synergistically, in both MGMT-unmet (BT453 and BT314; for proliferation −21.2% and −14.7%, respectively; for cell mortality +54% and +45%, respectively) and MGMT-met neurospheres (BT302 and BT138; for proliferation −20.2% and −10.31% and for cell mortality +66% and +33%, respectively). The effect on proliferation after TMZ treatment was observed for all the single miRNA signature members (Supplementary Fig. S2), but it was more pronounced when the entire signature was overexpressed in combination. Of note, the negative impact of miR-1/-26a-1/-487b signature on proliferation and cell viability was also evident after RT treatment (Supplementary Fig. S3).

### The miRNA signature induces cell death by necroptosis after TMZ treatment

**Fig. 2:**
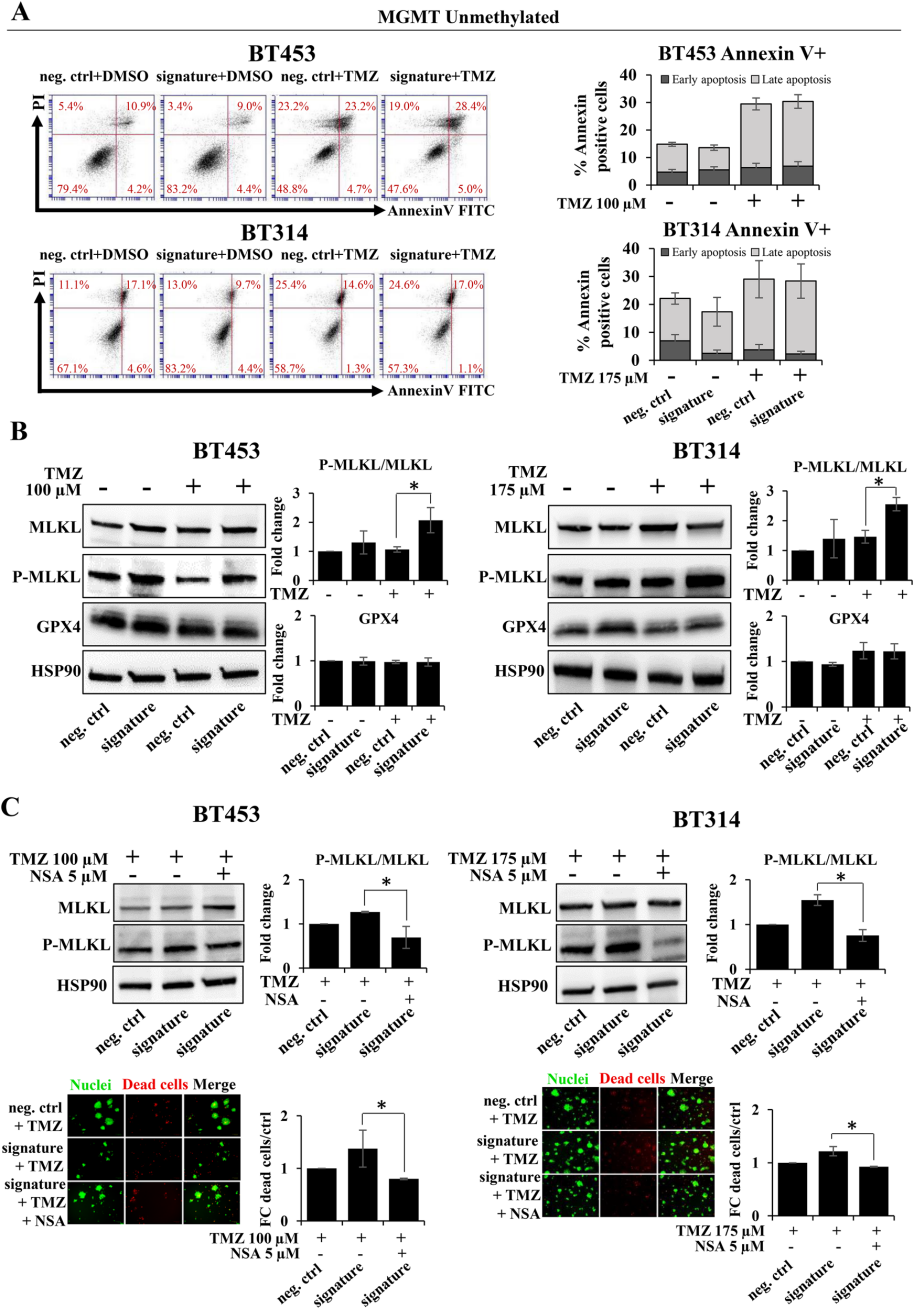
MiRNA signature effect on different types of cell death after TMZ treatment. **A** Analysis of Annexin V/PI by flow cytometry in the indicated GBM neurospheres overexpressing the miRNA signature mimics or negative control mimic (neg. ctrl) after 6 days of TMZ treatment with the indicated doses. *Upper panel*: representative experiment of the percentage of apoptotic cells. *Lower panel:* percentage of Annexin positive cells in TMZ-treated neurospheres overexpressing the miRNA signature compared to controls; **B** Western blots of a representative experiment of MLKL, p-MLKL expression (necroptosis-related markers) and GPX4 (ferroptosis-related marker) in the indicated GBM neurospheres overexpressing the miRNA signature with respect to control and treated with TMZ. **C** Analyses of cell viability by Cyto3D™Live-Dead assay and western blots of a representative experiment of MLKL and p-MLKL expression in the indicated GBM neurospheres overexpressing the miRNA signature and treated concomitantly with TMZ and NSA. Densitometric analyses by ImageJ software are shown. *p < 0.05; **p < 0.01.

Since MGMT-unmet patients are more resistant to TMZ and have much shorter survivals compared to those MGMT-met [7], we focused our study on the effects of the entire miR-1/-26a-1/-487b signature on MGMT-unmet GBM neurospheres. Thus, to further investigate the effect of the ectopic expression of miR-1/-26a-1/-487b signature on cell death, we analyzed cell cycle arrest and the induction of apoptosis after the TMZ treatment. Importantly, we did not observe neither alterations in the percentage of the cell cycle phases (data not shown) nor differences in terms of Annexin V+ cells in neurospheres overexpressing the miRNA signature after TMZ treatment (Fig. 2A). However, as shown in Fig. 1B, a significant increase, although not synergic, in dead cells is clearly evident after treatment in neurospheres overexpressing the miRNA signature. For this reason, after ectopic miRNA signature overexpression, we assessed other types of programmed cell death, such as necroptosis and ferroptosis [23]. Protein changes in Mixed lineage kinase domain-like 1 (MLKL) and phosphorylated-MLKL as markers of necroptosis, and glutathione peroxidase 4 (GPX4) as a central regulator of ferroptosis were examined. As shown in Fig. 2B, ectopic expression of the three-miRNA signature in TMZ-treated neurospheres induced a decrease of MLKL expression with an increase of phosphorylated-MLKL protein, whereas it did not produce any effects on GPX4 expression. To further strengthen that the miRNA signature is able to induce necroptosis during the treatment with TMZ, we performed rescue experiments with necrosulfonamide (NSA), a specific MLKL inhibitor that suppresses necroptosis. As shown in Fig. 2C we found that in TMZ-treated neurospheres overexpressing the miRNA signature, NSA inhibits MLKL activity and reduces cell death. Altogether, these data clearly demonstrate that the miRNA signature triggers cell death *via* necroptosis during TMZ treatment.

### FOXA1 and WT1 are novel and direct targets of the miR-1/-26a-1/-487b signature

**Fig. 3:**
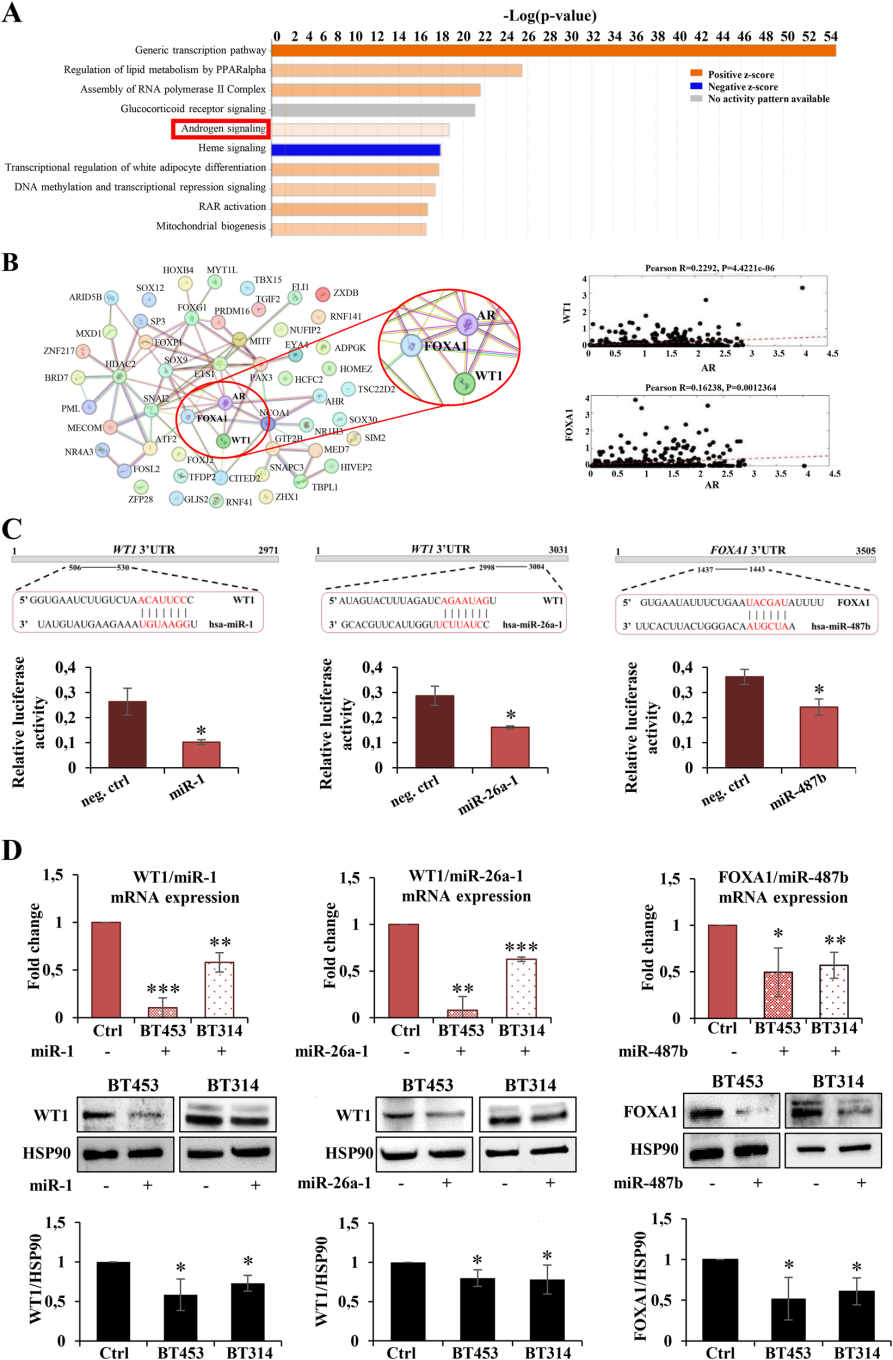
Identification and validation of WT1 and FOXA1 as miR-1-3p, miR-26a-1-3p, and miR-487b-3p direct targets. **A** Ingenuity pathway analysis by Qiagen tool of genes differentially expressed in TMZ-resistant compared to—sensitive GBM cells (GSE131781); **B** *Left panel:* Schematic representation of the predicted miRNA target genes interaction with androgen receptor (AR) by STRING analysis; *right panel*: scatter plots of the Pearson’s correlation of gene expression between WT1 and FOXA1 with AR in 1018 GBM patients from the CGGA RNAseq dataset. Correlation coefficient (*R*) and linear trend line are reported; **C** *Upper panel*: Schematic representation of putative binding sites of miR-1, miR-26a-1 in the 3′UTR of WT1 gene, and of miR-487b in the 3’UTR of FOXA1 gene. *Lower panel*: Firefly luciferase activity in recipient cells after transient co-transfection with Renilla luciferase reporter plasmid containing the 3′UTR of the indicated gene target sites, and the relative miRNA-mimic or ctrl-mimic. Results are expressed as fold activation relative to the basal activity of psiCHECK2 empty control; **D** WT1- and FOXA1-mRNA and related protein expression levels in the indicated GBM neurospheres after miRNAs overexpression. Densitometric analyses by ImageJ software are shown. All the values are reported as the mean of at least three experiments. Error bars indicate the standard deviation. *p ≤ 0.05, **p ≤ 0.01.

To identify the biological processes involved in TMZ response, we interrogated the GSE131781 GEO dataset and identified 216 genes upregulated in TMZ-resistant vs TMZ-sensitive cells (Fold change ≥2, *p* value ≤0.05) [24]. Then, we performed a pathway analysis of these genes using the IPA tool and we found the AR signaling pathway, whose aberrant activation promotes GBM progression and therapy resistance [24], as one of the top enriched pathways regarding TMZ response (Fig. 3A). To investigate which genes, among the identified 216, are impacted by the three-miRNA signature, a predictive target analysis using TargetScan was carried out. We found 53 genes as potential targets of the signature (Supplementary Table S1). By applying the STRING analysis to these putative targets, we found that two of them, the transcription factors Wilms tumor 1 (*WT1* as miR-1 and miR-26a-1 target) and Forkhead Box A1 (*FOXA1*; as miR-487b target), are direct activators of AR (Fig. 3B, *left panel*) [25, 26]. Lastly, a CGGA dataset (GBM patients, *n* = 1018) analysis showed that in patients, the expression of AR is positively correlated with both *WT1* and *FOXA1* (Fig. 3B, *right panel*). To assess whether the selected genes are direct targets of the miRNA signature, a human 3′UTR fragment for WT1 or FOXA1 transcripts was cloned downstream of the firefly luciferase reporter gene and co-transfected with the related miRNA mimics in HEK-293T recipient cells. The relative luciferase activity of the reporter with wild-type 3′UTR was decreased by 33% for miR-1, 44% for miR-26a-1 (WT1 3′UTR), and 61% for miR-487b (FOXA1 3′UTR) compared to their negative non-targeting control mimics. These data suggest that miR-1 and miR-26a-1 for WT1 and miR-487b for FOXA1 suppress their target gene expression through a direct interaction with their binding sequence in the 3′UTR (Fig. 3C). To further confirm these results, we evaluated the expression levels of WT1 and FOXA1 mRNAs and encoded proteins in MGMT-unmet neurospheres BT453 and BT314 overexpressing miR-1, miR-26a-1 or miR-487b. As shown in Fig. 3D*upper panel*, the mRNA levels of the selected targets are decreased upon miRNAs overexpression in both neurospheres. In agreement with these data, we also found a decrease in the respective protein levels (Fig. 3D*lower panel*).

### MiR-1/-26a-1/-487b signature impacts the androgen receptor pathway via WT1 and FOXA1 genes

**Fig. 4:**
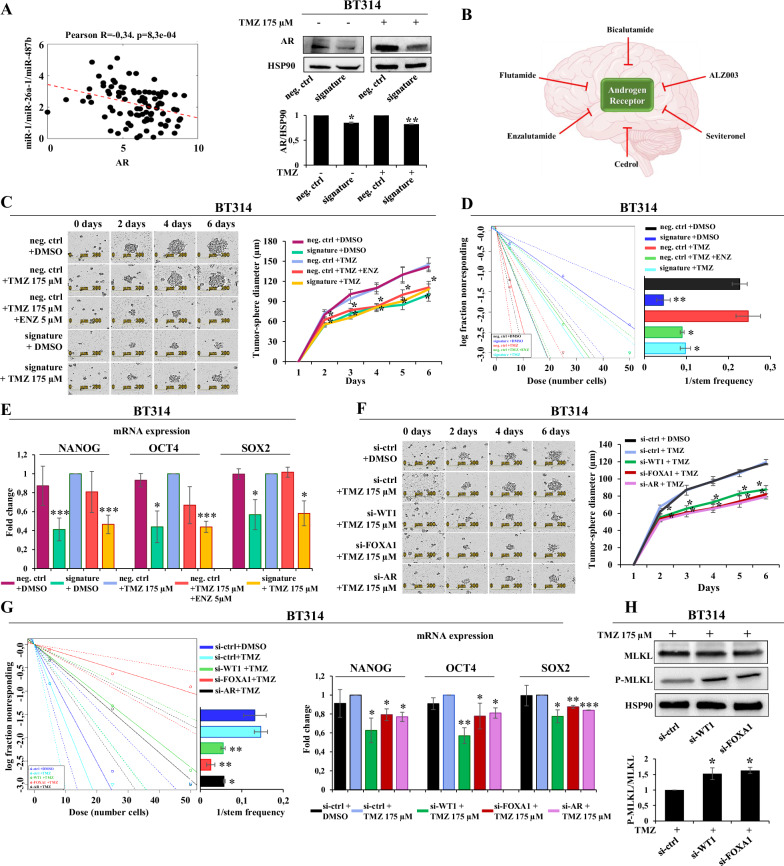
MiRNA signature impact on Androgen Receptor signaling by targeting WT1 and FOXA1. **A** *Left panel*: scatter plot of the Pearson’s correlation between the miRNA signature expression and AR in a TCGA miRNAseq dataset of 94 IDH wild-type glioma patients. Correlation coefficient (*R*) and linear trend line are reported; *Right panel*: Western blots of a representative experiment of AR expression in the indicated GBM neurospheres overexpressing the miRNA signature mimics or negative control mimic (neg. ctrl) after 6 days of TMZ treatment with the indicated doses. Densitometric analysis by ImageJ software is shown; **B** Schematic representation of AR inhibitors already evaluated on GBM; **C** IncuCyte time-course of tumor-spheres formation of TMZ- or ENZ-treated GBM cells after ectopic expression of the miRNA signature with respect to negative controls (neg. ctrl). Quantification of tumor-spheres diameter calculated by IncuCyte software; **D** Extreme limiting dilution analyses of TMZ- or ENZ-treated GBM cells after ectopic expression of the miRNA signature versus controls and graphic quantification of stem frequency. **E** mRNA expression levels of NANOG-, OCT4-, and SOX2- in TMZ-, or ENZ-treated GBM neurospheres overexpressing the miRNA signature compared to controls; **F** Tumor-spheres formation evaluated by IncuCyte time-course of TMZ- treated GBM cells after transfection of siRNAs- WT1 -FOXA1 or -AR versus controls. Quantification of tumor-spheres diameter calculated by IncuCyte software; **G** *Left panel*: Extreme limiting dilution analyses of TMZ- treated GBM cells after transfection of siRNAs- WT1 -FOXA1 or –AR versus controls and graphic quantification of stem frequency. *Right panel*: NANOG-, OCT4- and SOX2- mRNA expression levels in TMZ-treated GBM neurospheres after WT1, FOXA1, and AR downregulation by siRNA compared to controls. **H** Western blots of a representative experiment of MLKL and p-MLKL expression in TMZ-treated GBM neurospheres after WT1 and FOXA1 downregulation compared to controls. Densitometric analyses by ImageJ software are shown. *p ≤ 0.05, **p ≤ 0.01.

Targeting AR is a promising therapeutic strategy with the potential to abrogate treatment resistance in glioblastomas, as it is important for the maintenance and proliferation of cancer stem cells, thus fueling interest in the study of AR inhibitors, such as enzalutamide (ENZ), as treatment sensitizers [24]. Since the miR-1/-26a-1/-487b signature targets the WT1 and FOXA1 genes, which in turn control the AR signaling pathway, we first investigated the effect of the miRNA signature on AR. As shown in Fig. 4A*left panel*, in silico analysis showed that the miRNA signature expression was significantly anticorrelated with AR expression in a TCGA dataset (IDH wild-type glioma patients, *n* = 94). This inverse correlation was observed both in male (Pearson *R* = −0.32, *p* = 0.019) and in female (Pearson *R* = −0.38, *p* = 0.012) patients, thus being independent of biological sex (Supplementary Fig. S4). Moreover, the overexpression of the miRNA signature downregulated AR expression protein in MGMT-unmet neurospheres both alone and in combination with TMZ treatment (Fig. 4A*right panel*). Several AR inhibitors have been described in GBM (Fig. 4B). Importantly, we found that, after TMZ-treatment, the ectopic expression of the miRNA signature was able to impair the tumor-sphere forming ability (Fig. 4C), decrease spheroid frequency (Fig. 4D), as well as the AR inhibitor ENZ. Moreover, ectopic expression of the miRNA signature reduced the mRNA levels of the stem markers NANOG, OCT4 and SOX2 in MGMT-unmet neurospheres, with a higher effect compared to ENZ treatment (Fig. 4E). These effects of the miRNA signature are associated with a decrease of WT1 and FOXA1 expression elicited both by the miRNA signature alone and in combination with TMZ (Supplementary Fig. S5). To confirm that the effect of the miRNA signature on AR is mediated by WT1 and FOXA1, we evaluated the effects of their downregulation by siRNAs transfection. WT1 and FOXA1 silencing impaired the tumor-sphere forming ability of neurospheres treated with TMZ (Fig. 4F), decreased the spheroid frequency and reduced the mRNA expression of the stem markers NANOG, OCT4 and SOX2 (Fig. 4G*left and right panels*). Moreover, to further demonstrate that the stemness impairment produced by ENZ is due to the direct inhibition of AR and not to possible off-targets effects, we also evaluated GBM stemness after the direct silencing of AR and we found an impaired tumor-sphere forming ability, decreased spheroid frequency and reduced stem markers expression as observed after miRNA signature overexpression or WT1 and FOXA1 silencing (Fig. 4F, G). Necroptosis induction contributes to suppressing therapeutic resistance in cancer stem cells [27–29], thus we evaluated whether the identified targets WT1 and FOXA1, which have an impact on stemness by targeting AR, could also play a role in necroptosis activation. As demonstrated in Fig. 4H, WT1 and FOXA1 silencing produced a downregulation of total MLKL protein with a concomitant increase of its phosphorylated form in neurospheres treated with TMZ, recapitulating the effects elicited by the miRNA signature as shown in Fig. 2B.

## Discussion

Glioblastomas are among the most relentless primary intracranial tumors with an average survival expectancy of only 12–18 months [30]. The lack of therapeutic options and the rapid onset of resistance to the currently applied treatments are one of the reasons accounting for the dismal prognosis of GBM patients. Thus, a more profound understanding of the underlying molecular mechanisms governing chemoresistance has been a central focus of translational research efforts, in order to overcome the limitations that affect the currently available treatment protocols as well as to identify novel potential therapeutic targets [31].

Non-coding RNAs, particularly miRNAs, have been extensively studied as potential modulators of drug response in GBM and have been demonstrated to act both as sensitizers and as promoters of chemoresistance, having an impact on a number of biological processes and signaling pathways [32]. However, only few studies have investigated how the interplay of multiple miRNAs in a signature may outdo the effects of the single miRNA molecules [33] and enhance the therapeutic response of GBM. In this study, we demonstrate that a three-miRNA signature, with an oncosuppressive function in GBM [13], has a sensitizing action towards TMZ, reducing cell proliferation and viability upon treatment in GBM neurospheres, particularly in those with an unmethylated-MGMT status that display a major basal resistance to this drug. We further demonstrate that this reduced viability is due to the miRNA signature's impact on necroptosis, a particular form of programmed cell death that has been described to mediate response to chemotherapy in GBM [34].

To delve deeper into the molecular mechanisms regulated by the miRNA signature, we looked for potential target genes also deregulated on the basis of GBM response to TMZ, and we found WT1 and FOXA1 as novel direct targets of the three miRNAs. WT1 was first identified as an oncosuppressor in the Wilms’ tumors, but subsequent studies demonstrated its oncogenic function in several solid and hematologic malignancies [35, 36]. This gene encodes for a transcription factor regulating cell growth, differentiation, apoptosis and epithelial-to-mesenchymal transition [36]. WT1 participates in embryogenesis, as it is important for the development of multiple organs, as well as its involvement in mammalian sex determination [37]. Being expressed in several tumors, WT1 has been included among the main tumor antigens considered in developing cancer vaccines. Indeed, phase I/II clinical trials have demonstrated promising results for WT1 peptide vaccine immunotherapy in several tumors, including GBM [35].

FOXA1 belongs to a sub-family of the forkhead box (FOX) proteins, which act both as classic transcription factors and as pioneer factors by interacting with chromatin and promoting the recruitment of other transcription regulators [22]. FOXA1 is functionally required for the normal development of organs, including the brain, where it is involved in the maturation of dopamine neurons [22]. It has also a role in the tumorigenesis of several malignancies, including GBM, where it has been found to be overexpressed and to regulate cell cycle progression [38, 39]. Interestingly, both WT1 and FOXA1 are involved in the sex-hormone-regulated pathways and, in particular, in the AR signaling. In this context, WT1 may act as a regulator of AR expression [40] but also participates in the transcriptional activation of AR-regulated genes [41]. Similarly, FOXA1 cooperates in the regulation of AR downstream genes by acting as a pioneer factor for AR [42].

In analyzing the TCGA dataset of IDH wild-type glioma patients, we observed an inverse correlation between the expression of the three-miRNA signature and that of the AR. Furthermore, we found that the signature ectopic expression is able to downregulate AR in TMZ-treated GBM neurospheres. These results support the involvement of AR signaling in the biological effects elicited by the signature. Since AR is reported to regulate glioma stem cell maintenance [43], we investigated the impact of the miRNA signature on the GBM cell staminal features and found a downregulation of several stem markers elicited by the signature. All these results were recapitulated by the silencing of the WT1 and FOXA1 as well as by using the AR inhibitor ENZ, which further confirms that the functional impact of the miR-1/-26a-1/-487b signature on GBM response to TMZ is mediated by the WT1/FOXA1/AR axis.

An aberrant AR signaling has been described to promote GBM progression and resistance to therapy [24]. Thus, interfering with the AR pathway and/or inhibiting androgen synthesis are considered potential strategies for treating GBM. AR antagonists such as ENZ have been found to cross the blood-brain-barrier; thus, their antitumoral potential in GBM has been investigated, revealing that antiandrogens slow down the growth and radiosensitize AR-positive GBM cell lines and xenografts [44]. However, studies aimed at evaluating adverse clinical impacts of AR inhibitors in prostate cancer patients evidenced a number of adverse events associated with the Central Nervous System functions, such as amnesia, unspecified cognitive disorders, memory impairment and state of confusion [45].

Thus, the identification of alternative ways to impair AR signaling, such as that mediated by the miR-1/-26a-1/-487b signature, could be of crucial relevance for developing complementary therapies based on the inhibition of AR signaling in GBM patients. The application of AR inhibition strategies for the treatment of GBM should also be considered in the context of sex-related differences in the GBM biologic and molecular features. A higher incidence of GBM, together with a shorter survival, has been observed in males than in females, suggesting biological sex-associated differences in the pathogenesis of these tumors [46]. However, considering the AR signaling, its overexpression has been observed in the majority of GBM tumors compared to normal samples independently of the biological sex [47]. Also, an association between AR expression and glioma grade progression has been observed irrespective of biological sex [48]. Furthermore, we didn’t found differences in the miRNA signature-AR correlation between tumors from male and female patients included in the TCGA dataset. These results may indicate that both men and women could benefit from the inhibitory effect on AR signaling elicited by the signature in GBM. In any case, further investigation of the AR role in male and female GBMs is needed for the application of AR silencing therapeutic approaches for these tumors.

In conclusion, this study might support the potential application of the miR-1-3p, miR-26a-1-3p and miR-487b-3p signature as a sensitizer to therapy in GBM, paving the way for miRNA-based complementary therapies that are valuable for managing patients. The validation of these data in preclinical models, together with a safety assessment of the potential side effects of a miRNA-based therapeutic approach, is essential for the translational application of these results into the clinical setting.

## Material and methods

### Ethics approval and consent to participate

Neurospheres, obtained at IRCCS Candiolo Cancer Institute, were derived from glioblastomas surgically removed from patients enrolled at Fondazione IRCCS Istituto Neurologico Carlo Besta (Milan, Italy) [49], according to protocols approved by the Ethical Committee of the Institute. Informed written consent was obtained from all patients and studies were conducted according to the Declaration of Helsinki.

### Cell culture and treatments

BT453, BT314, BT302, and BT138 neurospheres were cultured in Dulbecco’s modified Eagle medium F12-Nutrient Mixture (Gibco, Thermo Fisher Scientifics, USA) supplemented with 2% B-27 Supplement Minus Vitamin A (Gibco, Thermo Fisher Scientifics, USA), 20 ng/mL of animal-free recombinant human EGF and recombinant human FGF-b (PeproTech, Thermo Fisher Scientifics, USA), and 1% penicillin–streptomycin (Gibco, Thermo Fisher Scientifics, USA). The cells were maintained in a 5% CO_2_ incubator at 37 °C. For treatments, neurospheres were exposed to TMZ (MERCK Calbiochem, USA), ionizing irradiation (IBL 437C, Schering, Germany), ENZ (Selleckchem, USA), or NSA (Selleckchem, USA) at a dose selected based on IC20 from dose-response curves (TMZ: range 5–175 µM; RT: range 2–6 Gy; ENZ: range 5–10 µM; NSA: 5 µM).

### Transfection and luciferase assays

For transient overexpression of miRNAs, neurospheres were transfected with single or combined miR-1-3p, miR-26a-1-3p and miR-487b-3p mimics or negative control mimics (Dharmacon, USA) using TransIT-X2 Dynamic Delivery System (Mirus Bio, USA) according to the manufacturer’s instructions. For transfections with the combined miRNA-mimics, a final concentration of 10 nM was used for each one, reaching a total 30 nM concentration of the combination of the three-miRNA mimics. Conversely, to assess the functional effects of each single miRNA, 10 nM of the miRNA-mimic of interest was used together with 20 nM of negative control in order to reach the same total oligonucleotide concentration.

For gene silencing, neurospheres were transfected with TransIT-X2 Dynamic Delivery System (Mirus Bio, USA) using 25 nM of WT1, FOXA1, or AR On Target Plus siRNA SMARTpool or negative control (Dharmacon, USA) according to the manufacturer’s instructions. SiRNA sequences are reported in the Supplementary Table S2.

For the Luc assay, fragments of 3′-UTR containing the seed sequences for miR-1-3p and miR-26a-1-3p in human WT1 gene (NM_024426) and for miR-487b-3p in FOXA1 (NM_004496) were cloned into the psiCHECK2 vector (Promega, USA) downstream of the luciferase gene. HEK-293T cells were co-transfected by Lipofectamine 3000 (Invitrogen, Thermo Fisher Scientifics, USA), with 400 ng of firefly luciferase reporter plasmid containing wild-type 3′UTRs of WT1 for miR-1, miR-26a-1, or FOXA1 for miR-487b. Forty-eight hours post-transfection, cells were lysed and luciferase activity quantified using the Dual Luciferase Reporter kit (Promega, USA), according to the manufacturer’s instructions.

### RNA extraction, reverse transcription and quantitative real-time PCR (RT‑qPCR)

Total RNA was extracted from neurospheres by the TRIsure reagent (Bioline, Meridian Bioscience, USA) and quantified by the NanoDrop ND-1000 spectrophotometer (Thermo Fisher Scientific, USA). Analysis of mature miRNAs was performed using TaqMan® MicroRNA Reverse Transcription Kit (Applied Biosystems, Thermo Fisher Scientifics, USA) followed by RT-qPCR according to the manufacturer’s instructions. Data were normalized to RNU44 small RNA. RNA quantification of WT1, FOXA1, SOX2, OCT4, and NANOG was performed using SYBR Green-based RT-qPCR as described [13] using specific primers, available upon request. GAPDH gene expression was used as an endogenous control. SYBR Green and TaqMan qPCR were run in the QuantStudio 6 Flex Detection system (Applied Biosystems, Thermo Fisher Scientifics, USA).

### Functional assays

Proliferation was assessed using the 3-(4,5-dimethylthiazol-2-yl)-2,5-diphenyltrazolium bromide (MTT) assay (Sigma, USA). Briefly, MTT solution was added to the treated and transfected cells. After 3 h at 37 °C, isopropanol solution was added. The absorbance was measured at 570 nm (microplate reader; ThermoScientific™ Multiskan™ FC Microplate Photometer).

Cell viability was evaluated with Cyto3D^TM^Live-Dead Assay Kit (TheWell Bioscience, USA) following the manufacturer’s instructions. Several images were taken using a Bio-Rad ZOE fluorescent cell imager under a phase contrast microscope (Bio-Rad, USA).

Flow cytometric analyses (BD Accuri C6^TM^, BD Biosciences, USA) were performed to evaluate the apoptosis induction by using the Apoptosis detection kit FITC (Invitrogen, Thermo Fisher Scientifics, USA) following the manufacturer’s instructions.

Tumor-spheres forming ability was evaluated by measuring the growth of the sphere diameter. Briefly, cells were plated in a 96-well plate at a low density and maintained at 37 °C under 5% CO_2_ in the IncuCyte incubator (Sartorius, USA) for 6 days. The cells were monitored every day by real-time monitoring and the diameter was measured using the IncuCyte software.

Extreme limiting dilution analyses (ELDA) were performed as reported [50]. Briefly, neurospheres were dissociated, transfected, and plated in suspension media at decreasing cellular densities (50, 25, and 5). Wells were imaged 7 days after plating and the number of wells with one or more sphere, greater than ~20-μm diameter, were scored. Data were analysed using the online ELDA software (http://bioinf.wehi.edu.au/software/elda/).

### Western blot

Extracts from transfected cells were subjected to immunoblot analysis as described [51]. Proteins of interest were detected using: anti-MLKL (Cell Signaling, USA), anti-P-MLKL (Cell Signaling, USA), anti-GPX4 (Sigma-Aldrich, USA), anti-WT1 (Cell Signaling, USA), anti-FOXA1 (Cell Signaling, USA), and anti-AR (Dako Agilent, Denmark) antibodies. Anti-HSP90 (Cell Signaling, USA) was used to normalize.

### Bioinformatic and statistical analysis

The GSE131781 dataset was used to investigate differentially expressed genes between the TMZ-resistant and TMZ-sensitive GBM cells [52]. Gene target prediction of selected miRNAs was defined by using the TargetScan web tool (https://www.targetscan.org/vert_80/). The Ingenuity Pathways Analysis (IPA, Qiagen, Netherlands) tool was used to perform pathway analyses. The STRING (https://string-db.org/) analysis was used to construct a protein-protein interaction network using a confidence score greater than or equal to 0.700. Standardized TCGA data, including miRNA array expression, RNAseq gene expression and clinical data, were obtained from the Broad Institute TCGA Genome Data Analysis Center (10.7908/C11G0KM9). Clinical annotations were obtained from the cBioPortal (www.cbioportal.org). A second cohort of miRNAseq and RNAseq expression data was obtained from the Chinese Glioma Genome Atlas (CGGA, http://www.cgga.org.cn/, [53]). Differential expression of miRNAs between subgroups of samples were evaluated by the two-sided Wilcoxon rank-sum test. The Pearson’s correlation coefficient was evaluated between matched samples with miRNA\mRNA expression. The analyses were conducted with MATLAB R2023b. The significance of intergroup differences was estimated with the two-tailed Student’s *t*-test. All results are shown as the mean ± standard deviation of the mean (SD) of three independent experiments. Statistical significance was considered to be *p* ≤ 0.05.

## Supplementary information

Supplementary data

Original Data

## Data Availability

The data underlying all findings of this study are publicly available at https://gbox.garr.it. Images of the Western blots uncropped gels are reported in the Supplementary Data File 2.
