# Supplementary material for: Androgen receptor inhibition sensitizes glioblastoma stem cells to temozolomide by the miR-1/miR-26a-1/miR-487b signature mediated WT1 and FOXA1 silencing
Source: Cell Death Discov. 2025 May 21;11:248. doi: 10.1038/s41420-025-02517-6 (PMC12095541; doi:10.1038/s41420-025-02517-6)
Supplement: Supplementary file 1 — Supplementary data [file 41420_2025_2517_MOESM1_ESM.docx]

**Summary:**

- Figure S1: Dose-response TMZ curves for the selection of IC20 doses
- Figure S2: MiR-1/-26a-1/-487b signature impairs cell proliferation after TMZ treatment
- Figure S3: MiRNA signature affects cell proliferation and viability after RT treatment
- Figure S4: Correlation between the miRNA signature and AR expression separately by biological gender
- Figure S5: WT1 and FOXA1 expression after overexpression of the miRNA signature
- Supplementary Table 1: Predictive target genes of the miR-1/-26a-1/-487b signature members
- Supplementary Table 2: siRNA sequences

**
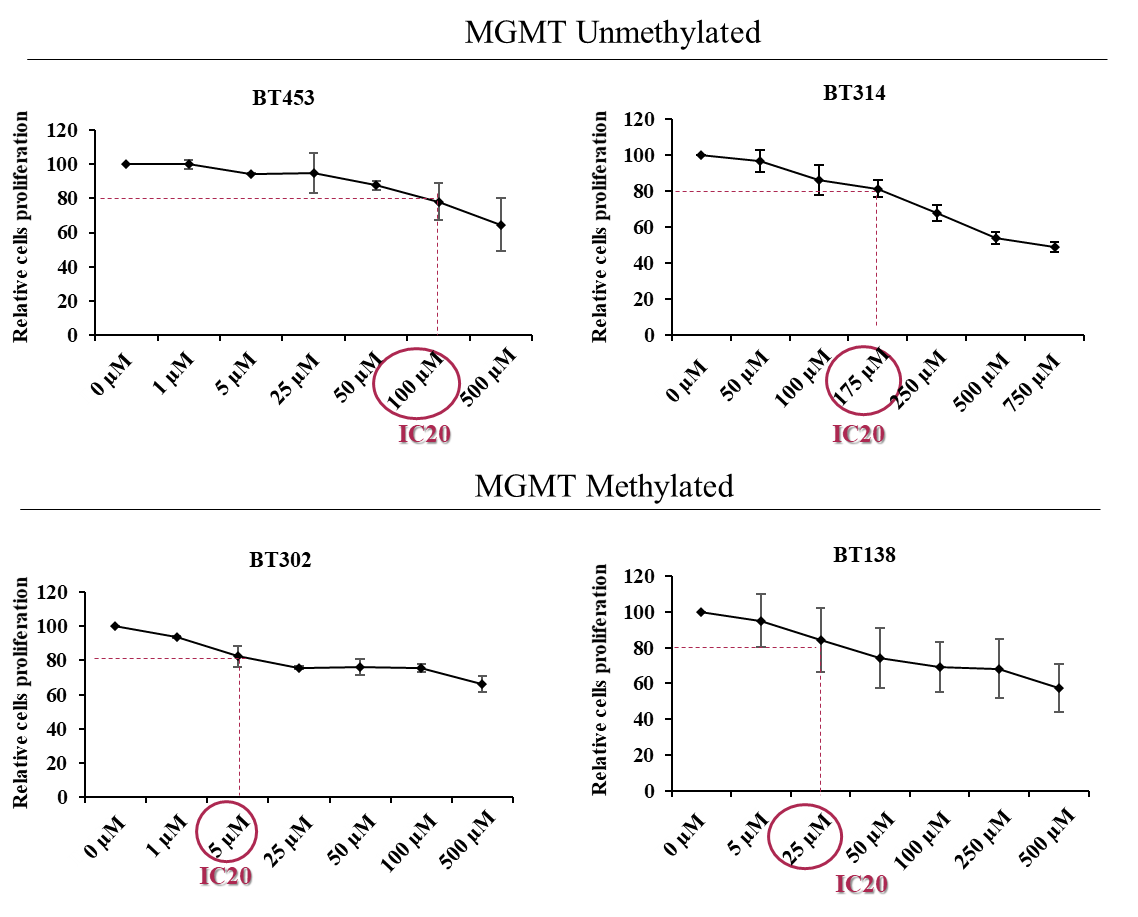
Figure S1**

**Figure S1: IC20 values for TMZ treatment in MGMT unmethylated and methylated GBM neurospheres.** Analysis of cell proliferation by MTT in the indicated GBM neurospheres to determinate the dose corresponding to IC20, comparing drug-treated groups against vehicle (DMSO) controls. Cell proliferation values are expressed as optical density at 750 nm wavelength, proportional to the quantity of metabolically active cells. All the values are reported as mean of at least three experiments. Error bars indicate the standard deviation.

**Figure S2**


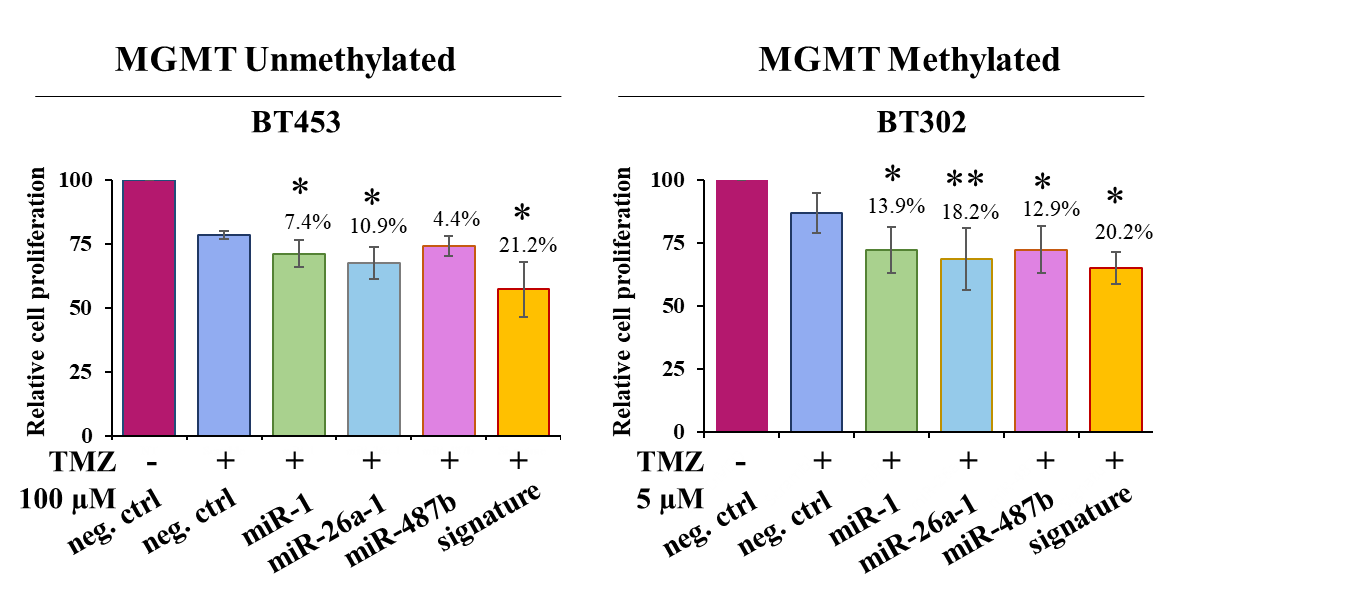


**Figure S2: MiR-1/-26a-1/-487b signature impairs cell proliferation after TMZ treatment.** Analysis of cell proliferation by MTT in the indicated GBM neurospheres overexpressing the miRNA signature mimics, single or in combination, or negative control mimic (neg. ctrl) after 6 days of TMZ treatment with the indicated doses. Cell proliferation values are expressed as optical density at 750 nm wavelength, proportional to the quantity of metabolically active cells. All the values are reported as mean of at least three experiments. Error bars indicate the standard deviation. * = p ≤0.05, ** = p ≤0.01.

**
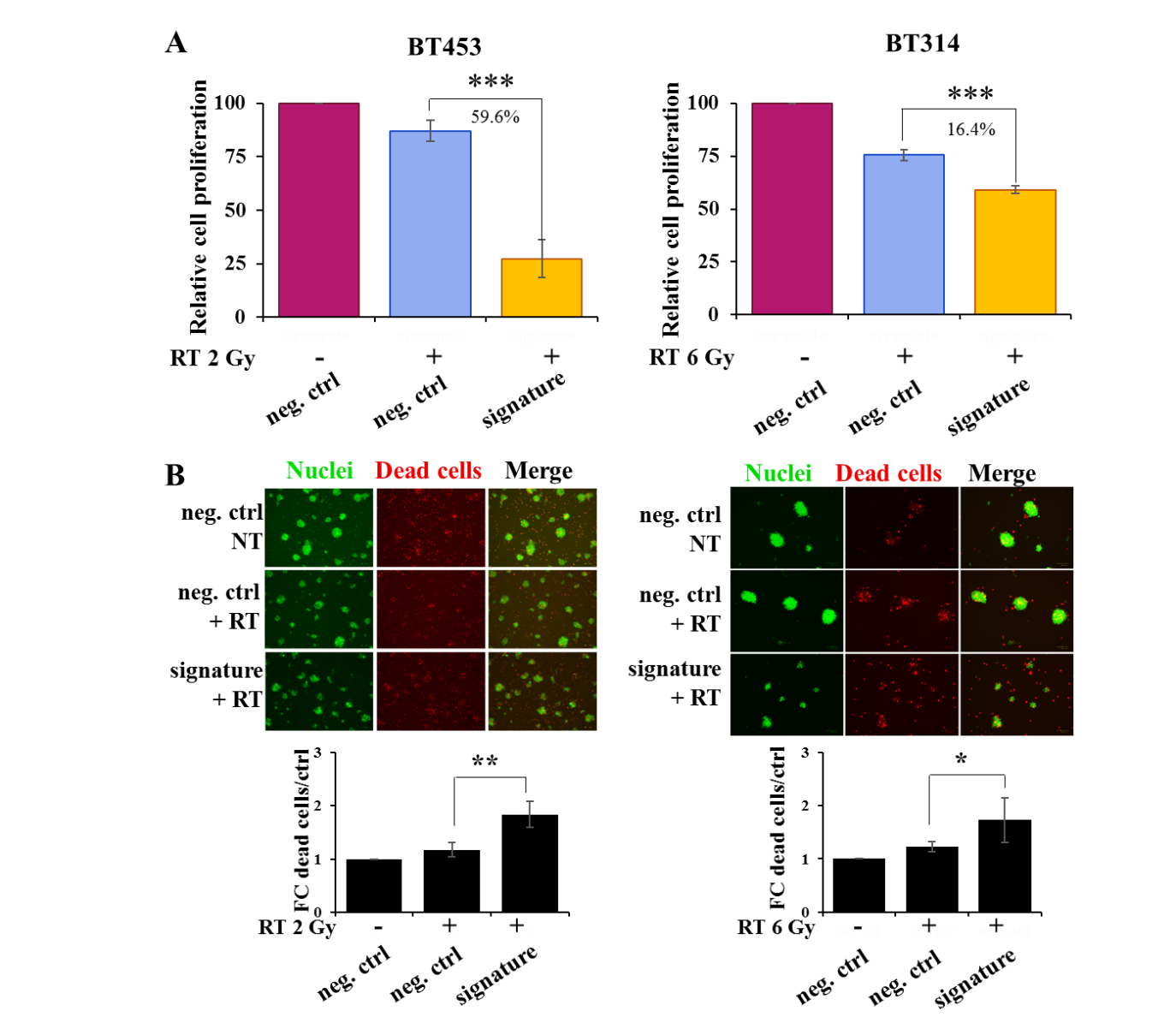
Figure S3**

**Figure S3: MiRNA signature affects cell proliferation and viability after RT treatment.** Analysis of (**a**) cell proliferation by MTT and (**b**) viability by Cyto3D™Live-Dead assay in the indicated GBM neurospheres overexpressing the miR-1/-26a-1/-487b signature mimics or negative control mimic (neg. ctrl) after 3 days of RT treatment with the indicated doses. Cell proliferation values are expressed as optical density at 750 nm wavelength, proportional to the quantity of metabolically active cells and viability as fold change of dead cells respect to control. All the values are reported as mean of at least three experiments. Error bars indicate the standard deviation. NT= non-treated; * = p ≤0.05, ** = p ≤0.01, *** = p ≤0.001.

**Figure S4**


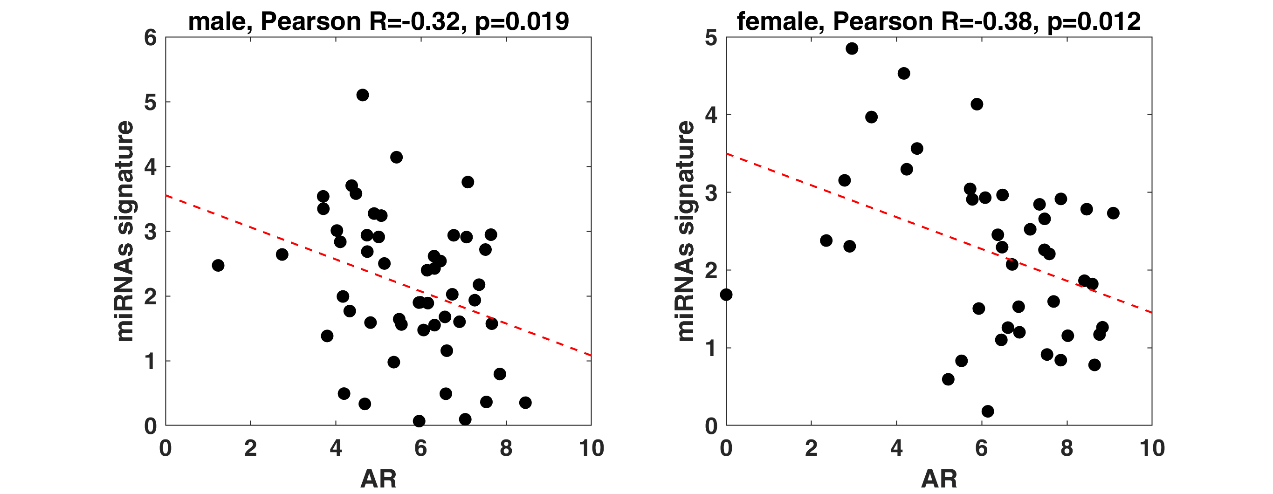


**Figure S4: The miRNA signature is significantly anticorrelated with AR expression independently of biological gender**. Scatter plot of the Pearson’s correlation between the miRNA signature expression and AR in a TCGA miRNA-seq dataset of 94 IDH wild-type glioma patients. Correlation coefficient (R) and linear trend line are reported.

**Figure S5**


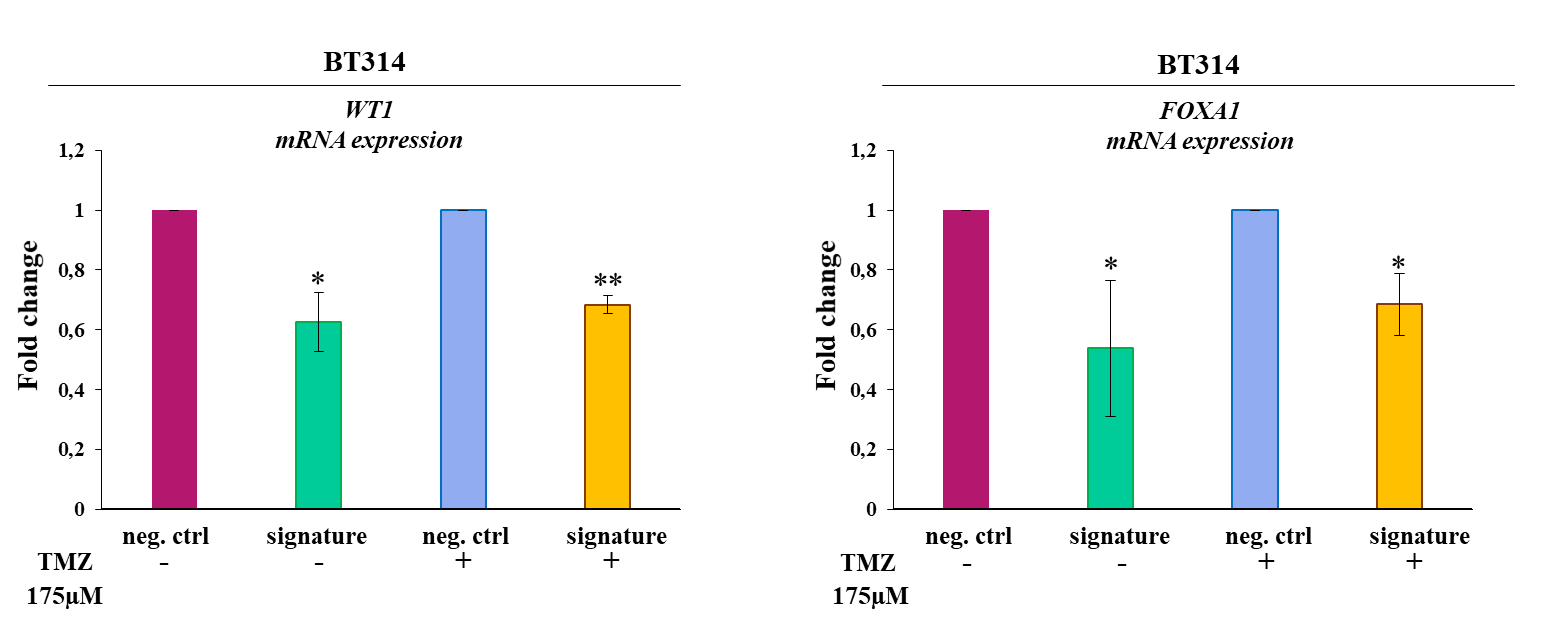


**Figure S5: The miRNA signature overexpression reduces the expression levels of WT1 and FOXA1**. WT1- and FOXA1-mRNA expression levels in the indicated GBM neurospheres after overexpression of the miR-1/-26a-1/-487b signature mimics or negative control mimic (neg. ctrl) after DMSO or TMZ treatment with the indicated doses. All the values are reported as mean of at least three experiments. Error bars indicate the standard deviation. * = p ≤0.05, ** = p ≤0.01.

**Supplementary Table 1. Predictive target genes of the miR-1/-26a-1/-487b signature members**

| **miR-1** | | |
| --- | --- | --- |
|  | **Fold change** | **P value** |
| **FOXP1** | 15,92 | 3,3E-06 |
| **PAX3** | 10,26 | 2,8E-05 |
| **SOX9** | 9,15 | 2,4E-06 |
| **HOXB4** | 7,83 | 8,8E-05 |
| **RNF141** | 7,33 | 3,7E-05 |
| **GLIS2** | 6,38 | 2,6E-06 |
| **FLI1** | 6,21 | 2,5E-04 |
| **NR4A3** | 5,03 | 7,4E-05 |
| **SIM2** | 3,93 | 1,1E-03 |
| **NR1H3** | 3,83 | 1,4E-06 |
| **HIVEP2** | 3,72 | 9,9E-06 |
| **SNAI2** | 3,36 | 4,6E-04 |
| **ADPGK** | 3,08 | 1,3E-05 |
| **TGIF2** | 2,72 | 4,8E-06 |
| **ATF2** | 2,38 | 1,5E-04 |
| **GTF2B** | 2,12 | 1,8E-03 |
| **ZNF217** | 2,10 | 4,3E-05 |
| **miR-26a-1** | | |
|  | **Fold change** | **P value** |
| **PRDM16** | 151,71 | 1,7E-06 |
| **CITED2** | 11,13 | 2,3E-08 |
| **ZHX1** | 7,67 | 1,4E-07 |
| **HCFC2** | 4,41 | 1,2E-05 |
| **FOXG1** | 3,96 | 2,9E-04 |
| **SNAPC3** | 3,95 | 9,5E-06 |
| **FOSL2** | 3,35 | 1,5E-06 |
| **FOXJ2** | 3,34 | 8,9E-06 |
| **MYT1L** | 3,31 | 4,0E-03 |
| **MITF** | 3,15 | 1,3E-06 |
| **TSC22D2** | 3,05 | 1,7E-06 |
| **SOX30** | 2,84 | 7,8E-04 |
| **SP3** | 2,77 | 9,8E-07 |
| **ARID5B** | 2,76 | 3,4E-05 |
| **TBX15** | 2,74 | 8,7E-06 |
| **PML** | 2,66 | 1,3E-04 |
| **HDAC2** | 2,57 | 3,4E-05 |
| **ZXDB** | 2,52 | 5,4E-06 |
| **ZFP28** | 2,51 | 3,1E-04 |
| **SOX12** | 2,50 | 1,2E-05 |
| **NUFIP2** | 2,46 | 1,3E-05 |
| **TFDP2** | 2,31 | 1,2E-04 |
| **BRD7** | 2,28 | 2,9E-04 |
| **MED7** | 2,25 | 2,7E-06 |
| **AHR** | 2,21 | 7,9E-05 |
| **TBPL1** | 2,04 | 7,1E-07 |
| **TP53** | 2,03 | 4,2E-04 |
| **miR-487b** | | |
|  | **Fold change** | **P value** |
| **FOXA1** | 8,93 | 1,2E-06 |
| **miR-1/miR-26a-1** | | |
|  | **Fold change** | **P value** |
| **WT1** | 85,58 | 2,1E-03 |
| **EYA4** | 3,76 | 7,6E-06 |
| **NCOA1** | 3,72 | 4,0E-03 |
| **MXD1** | 2,93 | 2,6E-07 |
| **MECOM** | 2,50 | 1,5E-04 |
| **ETS1** | 2,15 | 9,4E-05 |
| **miR-26a-1/miR-487b** | | |
|  | **Fold change** | **P value** |
| **HOMEZ** | 6,19 | 2,3E-04 |
| **RNF41** | 2,48 | 2,3E-05 |

**Supplementary Table 2. siRNA SMART pool sequences used for gene silencing experiments**

| siRNA | Code | Sequence |
| --- | --- | --- |
| **si-WT1** L-009101-00-0005 | J-009101-05 | CGAGAGCGAUAACCACACA |
|  | J-009101-06 | GCAGGAAGCACACUGGUGA |
|  | J-009101-07 | CUACAGCAGUGACAAUUUA |
|  | J-009101-08 | GGUCUGGCCGCAGCAAAU |
|  |  |  |
| **si-FOXA1** L-010319-00-0005 | J-010319-05 | GCACUGCAAUACUCGCCUU |
|  | J-010319-06 | CCUCGGAGCAGCAGCAUAA |
|  | J-010319-07 | GAACAGCUACUACGCAGAC |
|  | J-010319-08 | CCUAAACACUUCCUAGCUC |
|  |  |  |
| **si-AR** L-003400-00-0005 | J-003400-05 | GAGCGUGGACUUUCCGGAA |
|  | J-003400-06 | UCAAGGAACUCGAUCGUAU |
|  | J-003400-07 | CGAGAGAGCUGCAUCAGUU |
|  | J-003400-08 | CAGAAAUGAUUGCACUAUU |
|  |  |  |
