# Supplementary material for: Androgen receptor inhibition sensitizes glioblastoma stem cells to temozolomide by the miR-1/miR-26a-1/miR-487b signature mediated WT1 and FOXA1 silencing
Source: Cell Death Discov. 2025 May 21;11:248. doi: 10.1038/s41420-025-02517-6 (PMC12095541; doi:10.1038/s41420-025-02517-6)

Additional File – uncropped gels

Figure 2B: BT453

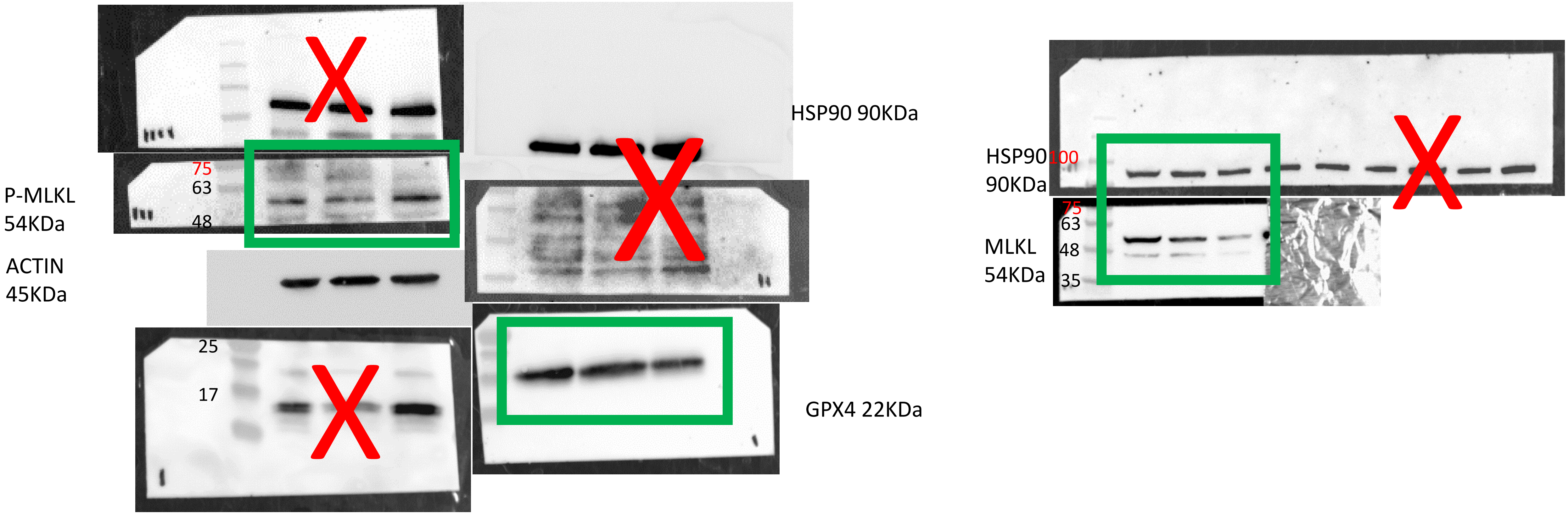

Figure 2B: BT314

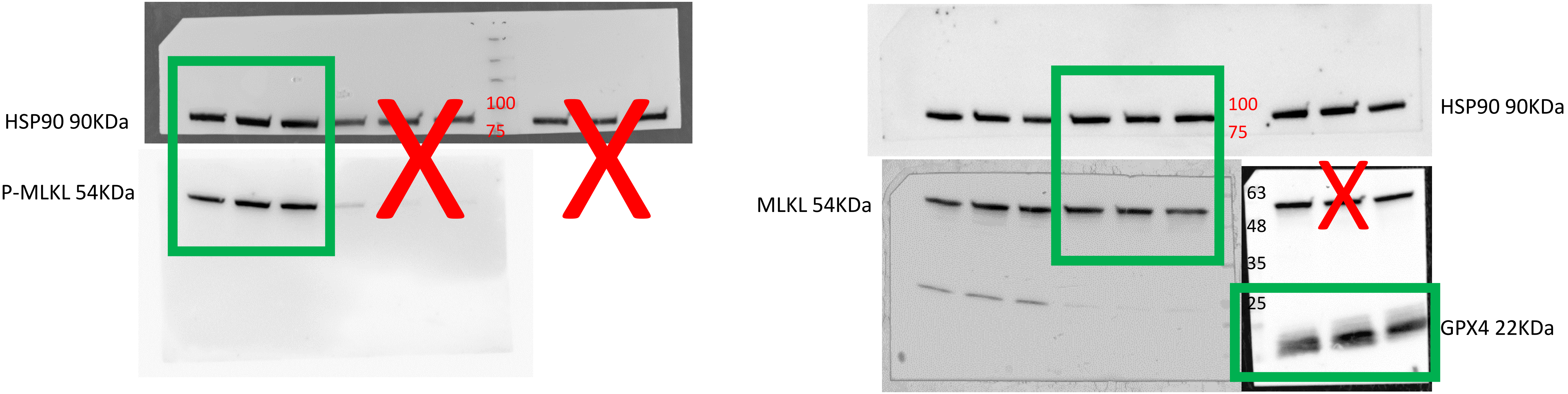

Figure 2B-C: BT453

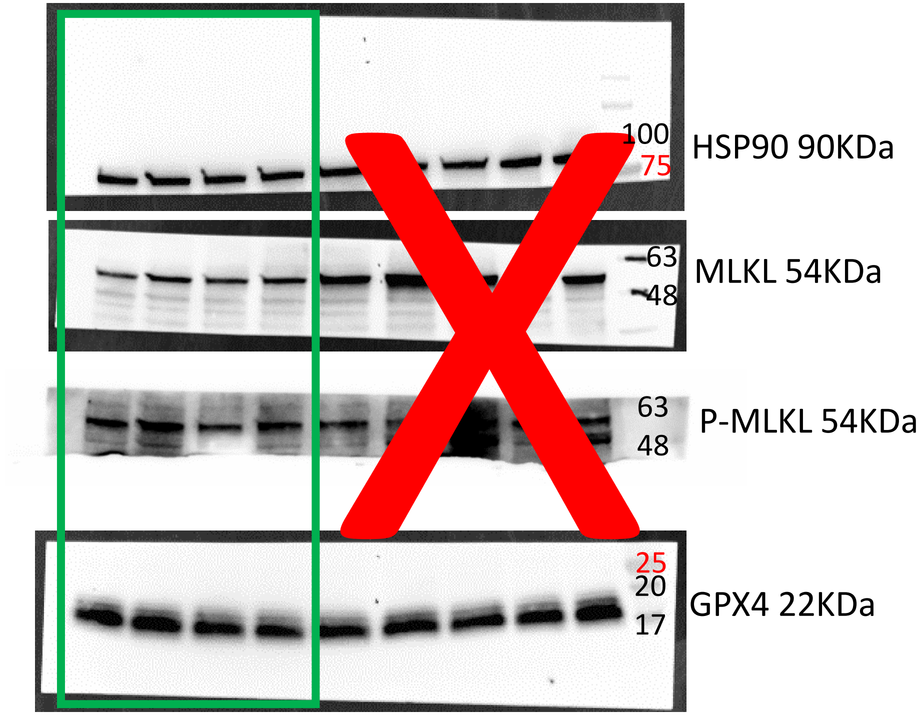

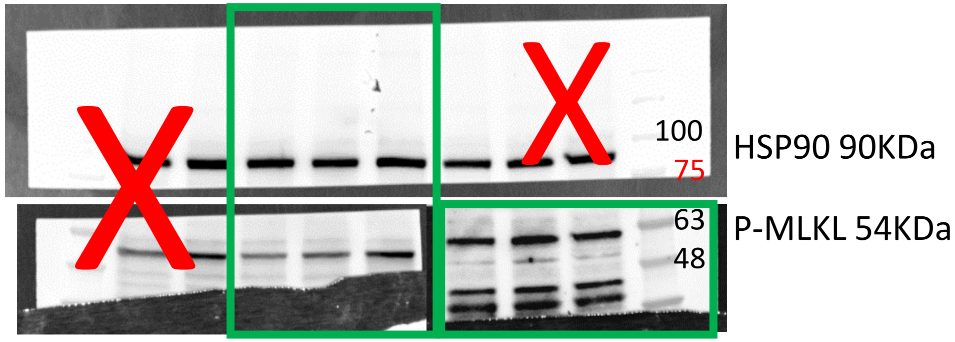

Figure 2B-C: BT314

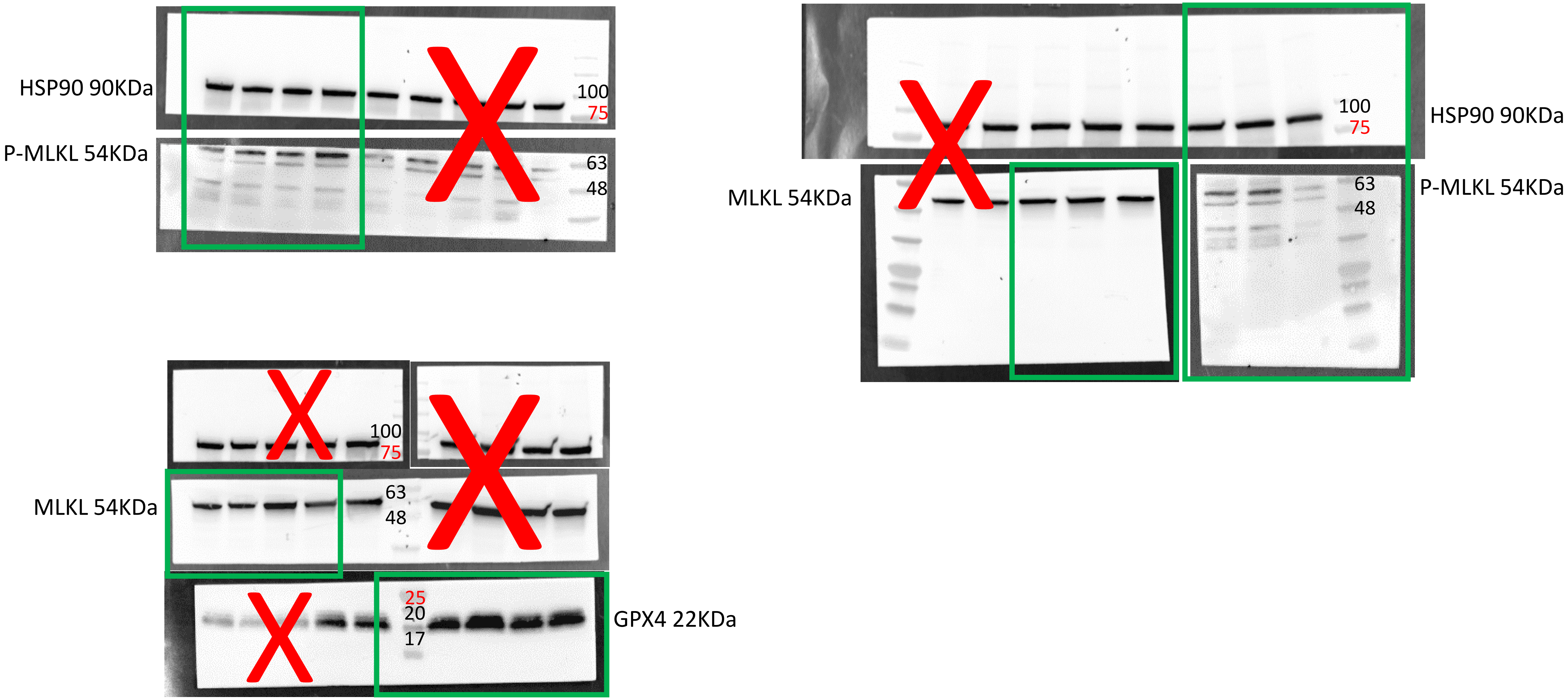

Figure 3D: BT453

WT1/miR-1

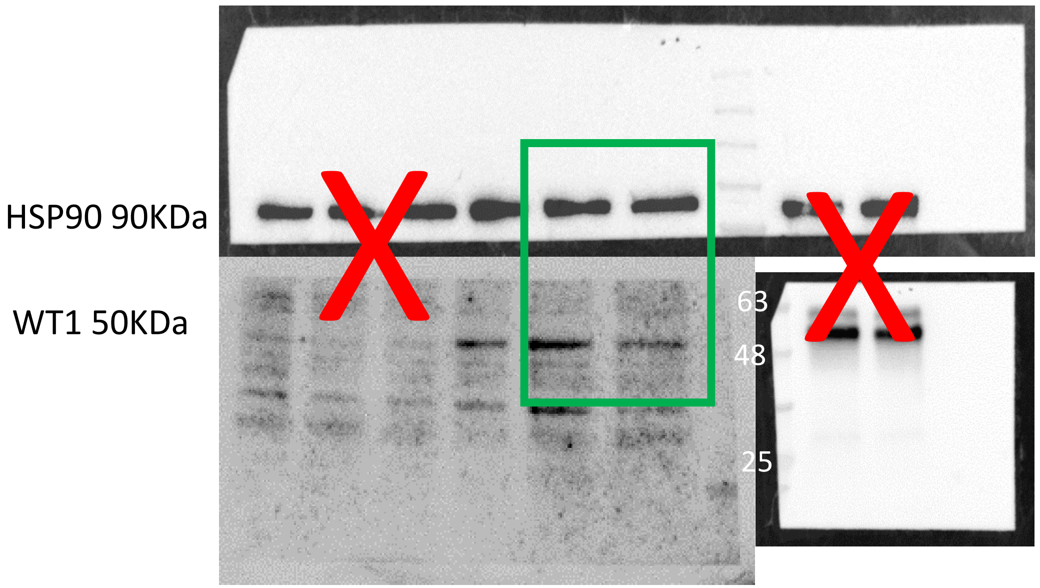

WT1/miR-26a-1

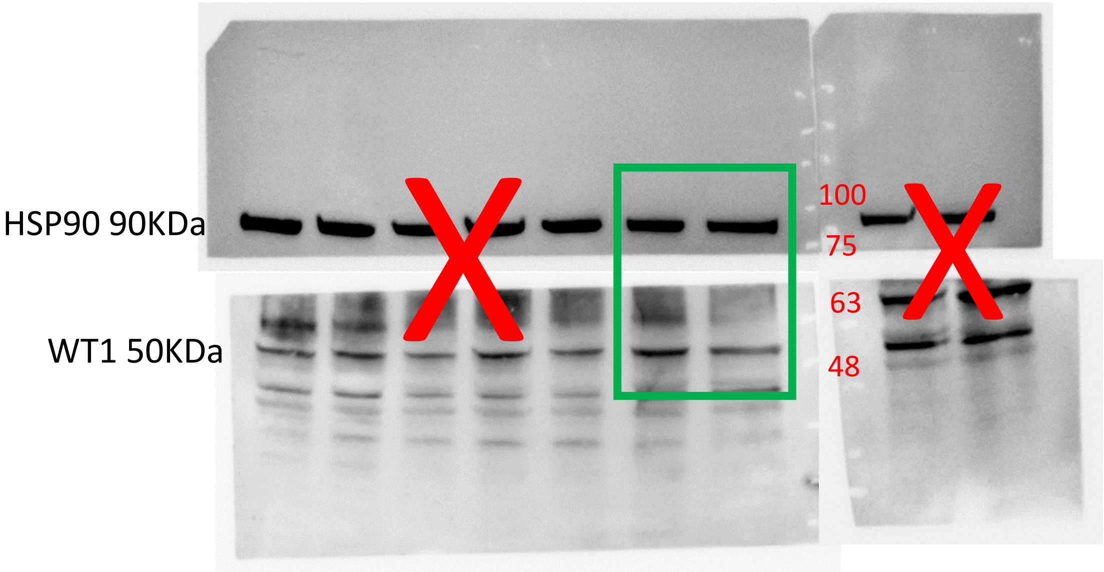

FOXA1/miR-487b

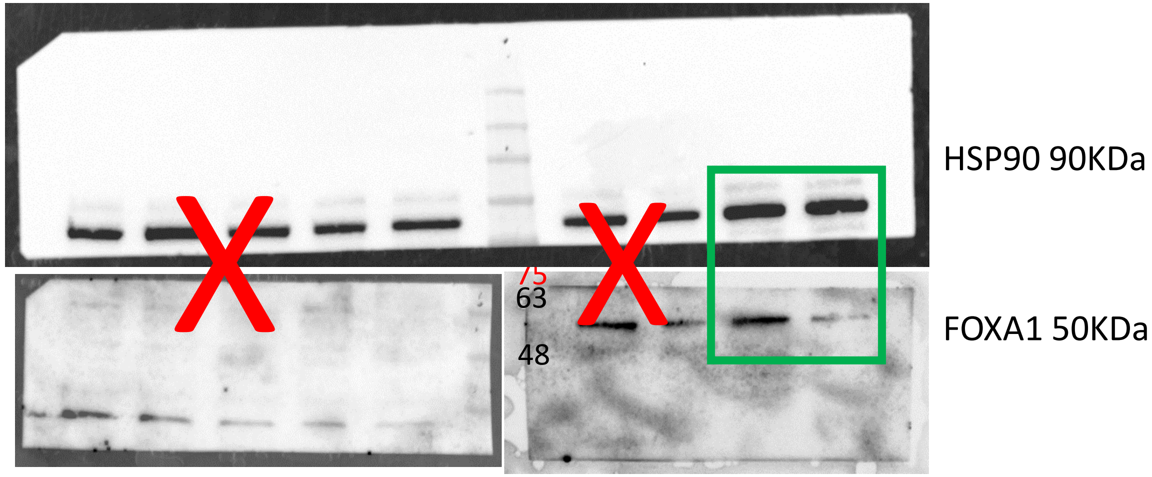

Figure 3D: BT314

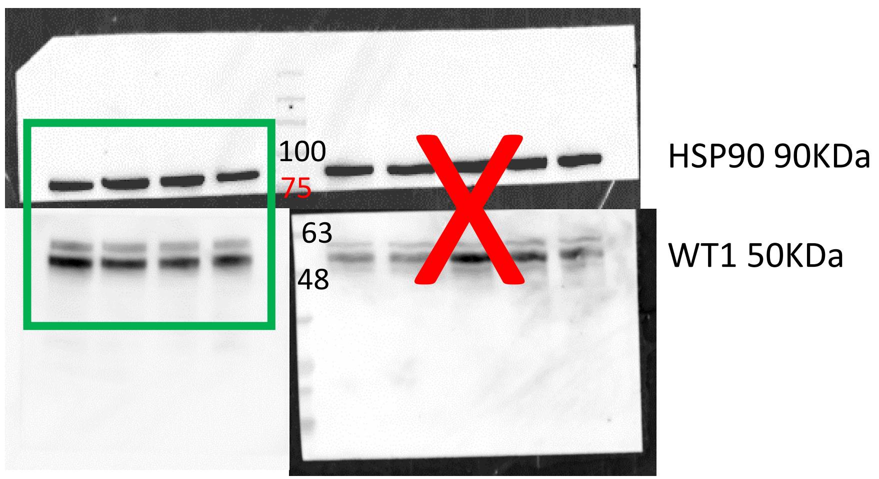

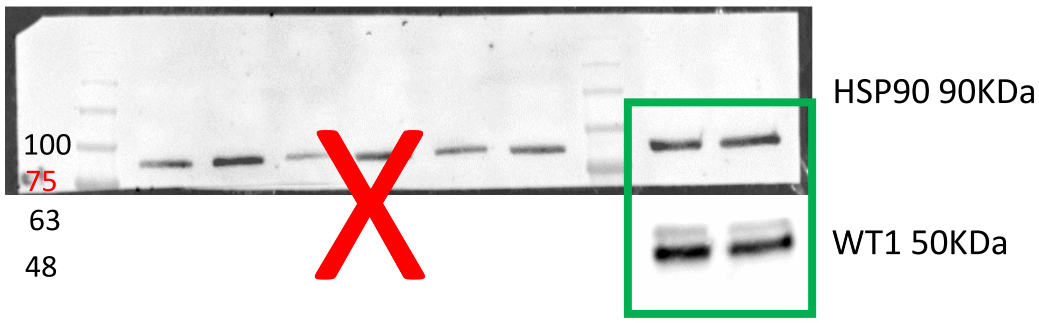

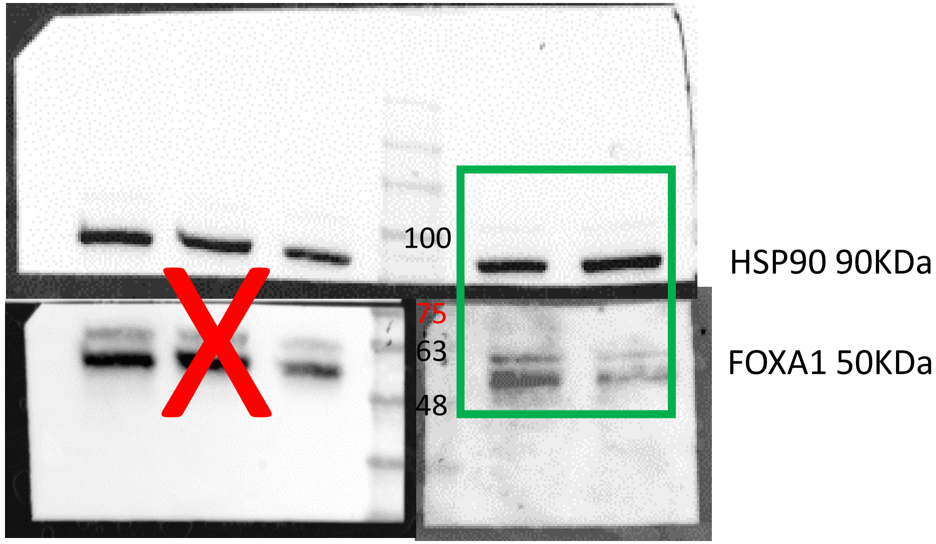

Figure 4A: BT314

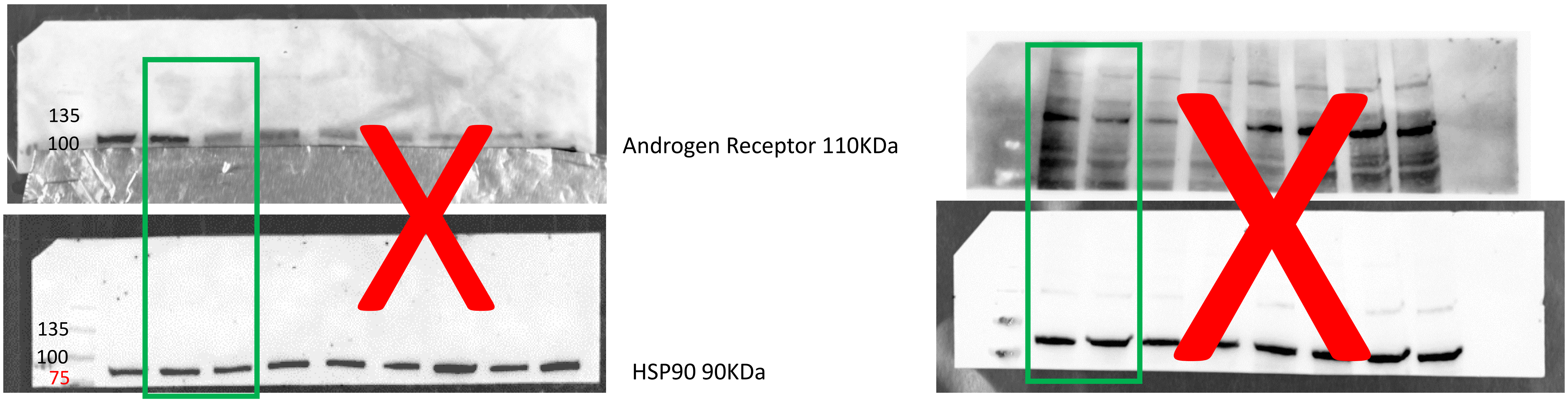

Figure 4H: BT314

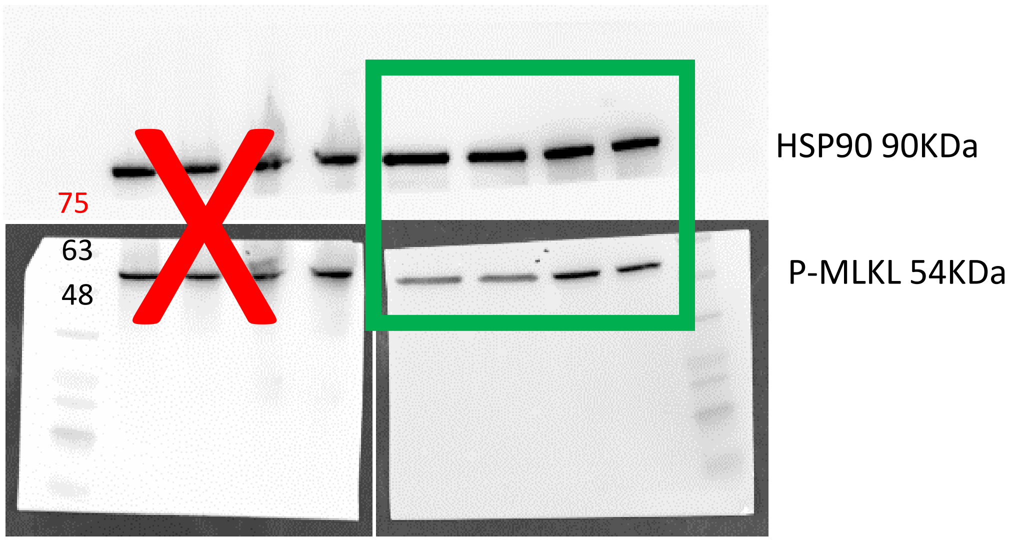

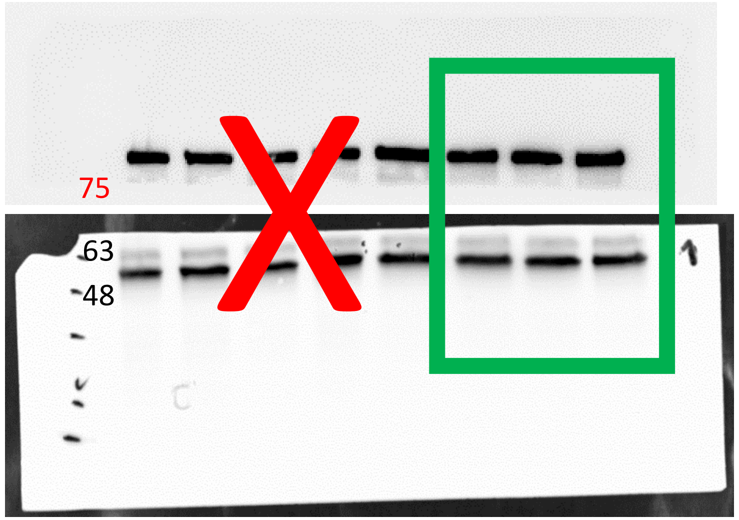

Supplement: Supplementary file 2 — Original Data [file 41420_2025_2517_MOESM2_ESM.pdf]
